# Unemployment among younger and older individuals: does conventional data about unemployment tell us the whole story?

**DOI:** 10.1186/s12651-018-0237-9

**Published:** 2018-03-08

**Authors:** Hila Axelrad, Miki Malul, Israel Luski

**Affiliations:** 1 Center on Aging & Work, Boston College, Chestnut Hill, MA 02467 USA; 2 The School of Social and Policy Studies, The Faculty of Social Sciences, Tel Aviv University, P.O. Box 39040, 6997801 Tel Aviv, Israel; 3 Department of Public Policy & Administration, Guilford Glazer Faculty of Business & Management, Ben-Gurion University of the Negev, Beer Sheva, Israel; 4 Department of Economics, The Western Galilee College, Akko, Israel

**Keywords:** Unemployment, Employment, Aging, Older workers, OECD, J64, J68

## Abstract

In this research we show that workers aged 30–44 were significantly more likely than those aged 45–59 to find a job a year after being unemployed. The main contribution is demonstrating empirically that since older workers’ difficulties are related to their age, while for younger individuals the difficulties are more related to the business cycle, policy makers must devise different programs to address unemployment among young and older individuals. The solution to youth unemployment is the creation of more jobs, and combining differential minimum wage levels and earned income tax credits might improve the rate of employment for older individuals.

## Introduction

Literature about unemployment references both the unemployment of older workers (ages 45 or 50 and over) and youth unemployment (15–24). These two phenomena differ from one another in their characteristics, scope and solutions.

Unemployment among young people begins when they are eligible to work. According to the International Labor Office (ILO), young people are increasingly having trouble when looking for their first job (ILO 2011). The sharp increase in youth unemployment and underemployment is rooted in long-standing structural obstacles that prevent many youngsters in both OECD countries and emerging economies from making a successful transition from school to work. Not all young people face the same difficulties in gaining access to productive and rewarding jobs, and the extent of these difficulties varies across countries. Nevertheless, in all countries, there is a core group of young people facing various combinations of high and persistent unemployment, poor quality jobs when they do find work and a high risk of social exclusion (Keese et al. 2013). The rate of youth unemployment is much higher than that of adults in most countries of the world (ILO 2011; Keese et al. 2013; O’Higgins 1997; Morsy 2012). Official youth unemployment rates in the early decade of the 2010s ranged from under 10% in Germany to around 50% in Spain (http://www.indexmundi.com/g/r.aspx?v=2229; Pasquali 2012). The youngest employees, typically the newest, are more likely to be let go compared to older employees who have been in their jobs for a long time and have more job experience and job security (Furlong et al. 2012). However, although unemployment rates among young workers are relatively higher than those of older people, the period of time they spend unemployed is generally shorter than that of older adults (O’Higgins 2001).

We would like to argue that one of the most important determinants of youth unemployment is the economy’s rate of growth. When the aggregate level of economic activity and the level of adult employment are high, youth employment is also high.^1^ Quantitatively, the employment of young people appears to be one of the most sensitive variables in the labor market, rising substantially during boom periods and falling substantially during less active periods (Freeman and Wise 1982; Bell and Blanchflower 2011; Dietrich and Möller 2016). Several explanations have been offered for this phenomenon. First, youth unemployment might be caused by insufficient skills of young workers. Another reason is a fall in aggregate demand, which leads to a decline in the demand for labor in general. Young workers are affected more strongly than older workers by such changes in aggregate demand (O’Higgins 2001). Thus, our first research question is whether young adults are more vulnerable to economic shocks compared to their older counterparts.

Older workers’ unemployment is mainly characterized by difficulties in finding a new job for those who have lost their jobs (Axelrad et al. et al. 2013). This fact seems counter-intuitive because older workers have the experience and accumulated knowledge that the younger working population lacks. The losses to society and the individuals are substantial because life expectancy is increasing, the retirement age is rising in many countries, and people are generally in good health (Axelrad et al. 2013; Vodopivec and Dolenc 2008).

The difficulty that adults have in reintegrating into the labor market after losing their jobs is more severe than that of the younger unemployed. Studies show that as workers get older, the duration of their unemployment lengthens and the chances of finding a job decline (Böheim et al. 2011; De Coen et al. 2010). Therefore, our second research question is whether older workers’ unemployment stems from their age.

In this paper, we argue that the unemployment rates of young people and older workers are often misinterpreted. Even if the data show that unemployment rates are higher among young people, such statistics do not necessarily imply that it is harder for them to find a job compared to older individuals. We maintain that youth unemployment stems mainly from the characteristics of the labor market, not from specific attributes of young people. In contrast, the unemployment of older individuals is more related to their specific characteristics, such as higher salary expectations, higher labor costs and stereotypes about being less productive (Henkens and Schippers 2008; Keese et al. 2006). To test these hypotheses, we conduct an empirical analysis using statistics from the Israeli labor market and data published by the OECD. We also discuss some policy implications stemming from our results, specifically, a differential policy of minimum wages and earned income tax credits depending on the worker’s age.

Following the introduction and literary review, the next part of our paper presents the existing data about the unemployment rates of young people and adults in the OECD countries in general and Israel in particular. Than we present the research hypotheses and theoretical model, we describe the data, variables and methods used to test our hypotheses. The regression results are presented in Sect. 4, the model of Business Cycle is presented in Sect. 5, and the paper concludes with some policy implications, a summary and conclusions in Sect. 6.

## Literature review

Over the past 30 years, unemployment in general and youth unemployment in particular has been a major problem in many industrial societies (Isengard 2003). The transition from school to work is a rather complex and turbulent period. The risk of unemployment is greater for young people than for adults, and first jobs are often unstable and rather short-lived (Jacob 2008). Many young people have short spells of unemployment during their transition from school to work; however, some often get trapped in unemployment and risk becoming unemployed in the long term (Kelly et al. 2012).

Youth unemployment leads to social problems such as a lack of orientation and hostility towards foreigners, which in turn lead to increased social expenditures. At the societal level, high youth unemployment endangers the functioning of social security systems, which depend on a sufficient number of compulsory payments from workers in order to operate (Isengard 2003).

Workers 45 and older who have lost their jobs often encounter difficulties in finding a new job (Axelrad et al. 2013; Marmora and Ritter 2015) although today they are more able to work longer than in years past (Johnson 2004). In addition to the monetary rewards, work also offers mental and psychological benefits (Axelrad et al. 2016; Jahoda 1982; Winkelmann and Winkelmann 1998). Working at an older age may contribute to an individual’s mental acuity and provide a sense of usefulness.

On average, throughout the OECD, the hiring rate of workers aged 50 and over is less than half the rate for workers aged 25–49. The low re-employment rates among older job seekers reflect, among other things, the reluctance of employers to hire older workers. Lahey (2005) found evidence of age discrimination against older workers in labor markets. Older job applicants (aged 50 or older), are treated differently than younger applicants. A younger worker is more than 40% more likely to be called back for an interview compared to an older worker. Age discrimination is also reflected in the time it takes for older adults to find a job. Many workers aged 45 or 50 and older who have lost their jobs often encounter difficulties in finding a new job, even if they are physically and intellectually fit (Hendels 2008; Malul 2009). Despite the fact that older workers are considered to be more reliable (McGregor and Gray 2002) and to have better business ethics, they are perceived as less flexible or adaptable, less productive and having higher salary expectations (Henkens and Schippers 2008). Employers who hesitated in hiring older workers also mentioned factors such as wages and non-wage labor costs that rise more steeply with age and the difficulties firms may face in adjusting working conditions to meet the requirements of employment protection rules (Keese et al. 2006).

Thus, we have a paradox. On one hand, people live longer, the retirement age is rising, and older people in good health want or need to keep working. At the same time, employers seek more and more young workers all the time. This phenomenon might marginalize skilled and experience workers, and take away their ability to make a living and accrue pension rights. Thus, employers’ reluctance to hire older workers creates a cycle of poverty and distress, burdening the already overcrowded social institutions and negatively affecting the economy’s productivity and GDP (Axelrad et al. 2013).

### OECD countries during the post 2008 crisis

The recent global economic crisis took an outsized toll on young workers across the globe, especially in advanced economies, which were hit harder and recovered more slowly than emerging markets and developing economies. Does this fact imply that the labor market in Spain and Portugal (with relatively high youth unemployment rates) is less “friendly” toward younger individuals than the labor market in Israel and Germany (with a relatively low youth unemployment rate)? Has the market in Spain and Portugal become less “friendly” toward young people during the last 4 years? We argue that the main factor causing the increasing youth unemployment rates in Spain and Portugal is the poor state of the economy in the last 4 years in these countries rather than a change in attitudes toward hiring young people.

OECD data indicate that adult unemployment is significantly lower than youth unemployment. The global economic crisis has hit young people very hard. In 2010, there were nearly 15 million unemployed youngsters in the OECD area, about four million more than at the end of 2007 (Scarpetta et al. 2010).

### Israel

From an international perspective, and unlike other developed countries, Israel has a young age structure, with a high birthrate and a small fraction of elderly population. Israel has a mandatory retirement age, which differs for men (67) and women (62), and the labor force participation of older workers is relatively high (Stier and Endeweld 2015), therefore, we believe that Israel is an interesting case for studying.

The Israeli labor market is extremely flexible (e.g. hiring and firing are relatively easy), and mobile (workers can easily move between jobs) (Peretz 2016). Focusing on Israel’s labor market, we want to check whether this is true for older Israeli workers as well, and whether there is a difference between young and older workers.

**Fig. 1:**
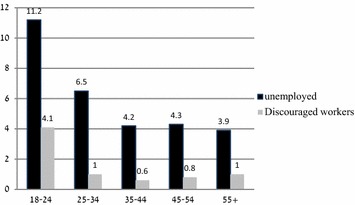
Unemployed persons and discouraged workers as percentages of the civilian labor force, by age group (Bank of Israel 2011). We excluded those living outside settled communities or in institutions. The percentages of discouraged workers are calculated from the civilian labor force after including them in it (Source: Calculated by the authors by using data from the Labor Force survey of the Israeli CBS, 2011)

The problem of unemployment among young people in Israel is less severe than in most other developed countries. This low unemployment rate is a result of long-term processes that have enabled the labor market to respond relatively quickly to changes in the economic environment and have reduced structural unemployment.^2^ Furthermore, responsible fiscal and monetary policies, and strong integration into the global market have also promoted employment at all ages. With regard to the differences between younger and older workers in Israel, Stier and Endeweld (2015) determined that older workers, men and women alike, are indeed less likely to leave their jobs. This finding is similar to other studies showing that older workers are less likely to move from one employer to another. According to the U.S. Bureau of Labor Statistics, the median employee tenure is generally higher among older workers than younger ones (BLS 2014). Movement in and out of the labor market is highest among the youngest workers. However, these young people are re-employed quickly, while older workers have the hardest time finding jobs once they become unemployed. The Bank of Israel calculated the chances of unemployed people finding work between two consecutive quarters using a panel of the Labor Force Survey for the years 1996–2011. Their calculations show that since the middle of the last decade the chances of unemployed people finding a job between two consecutive quarters increased.^3^ However, as noted earlier, as workers age, the duration of their unemployment lengthens. Prolonged unemployment erodes the human capital of the unemployed (Addison et al. 2004), which has a particularly deleterious effect on older workers. Thus, the longer the period of unemployment of older workers, the less likely they will find a job (Axelrad and Luski 2017). Nevertheless, as Fig. 1 shows, the rates of youth unemployment in Israel are higher than those of older workers.

We argue that the main reason for this situation is the status quo in the labor market, which is general and not specific to Israel. It applies both to older workers and young workers who have a job. The status quo is evident in the situation in which adults (and young people) already in the labor market manage to keep their jobs, making the entrance of new young people into the labor market more difficult. What we are witnessing is not evidence of a preference for the old over the young, but the maintaining of the status quo.

The rate of employed Israelis covered by collective bargaining agreements increases with age: up to age 35, the rate is less than one-quarter, and between 50 and 64 the rate reaches about one-half. In effect, in each age group between 25 and 60, there are about 100,000 covered employees, and the lower coverage rate among the younger ages derives from the natural growth in the cohorts over time (Bank of Israel 2013). The wave of unionization in recent years is likely to change only the age profile of the unionization rate and the decline in the share of covered people over the years, to the extent that it strengthens and includes tens of thousands more employees from the younger age groups.^4^

The fact that the percentage of employees covered by collective agreement increases with age implies that there is a status quo effect. Older workers are protected by collective agreements, and it is hard to dismiss them (Culpepper 2002; Palier and Thelen 2010). However, young workers enter the workforce with individual contracts and are not protected, making it is easier to change their working conditions and dismiss them.

**Fig. 2:**
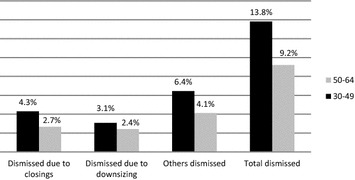
Dismissal of employees in Israel, by age. Percentage of total employed persons ages 20–75 and over including those dismissed (Source: Israeli Central Bureau of Statistics, 2008, data processed by the authors)

To complete the picture, Fig. 2 shows that the number of layoffs among adults is lower, possibly due to their protection under collective bargaining agreements.

In order to determine the real difference between the difficulties of older versus younger individuals in finding work, we have to eliminate the effect of the status quo in the labor market. For example, if we removed all of the workers from the labor market, what would be the difference between the difficulties of older people versus younger individuals in finding work? In the next section we will analyze the probability of younger and older individuals moving from unemployment to employment when we control for the status quo. We will do so by considering only individuals who have not been employed at least part of the previous year.

## Estimating the chances of finding a job and research hypotheses

Based on the literature and the classic premise that young workers are more vulnerable to economic shocks (ILO 2011), we posit that:

*H*_1_: The unemployment rate of young people stems mainly from the characteristics of the labor market and less from their personal attributes.

Based on the low hiring rate of older workers (OECD 2006) and the literature about age discrimination against older workers in labor markets (Axelrad et al. 2013; Lahey 2005), we hypothesis that:

*H*_2_: The difficulty face by unemployed older workers searching for a job stems mainly from their age and less from the characteristics of the labor market.

To assess the chances of younger and older workers finding a job, we used a logit regression model that has been validated in previous studies (Brander et al. 2002; Flug and Kassir 2001). Being employed was the dependent variable, and the characteristics of the respondents (age, gender, ethnicity and education) were the independent variables. The dependent variable was nominal and dichotomous with two categories: 0 or 1. We defined the unemployed as those who did not work at all during the last year or worked less than 9 months last year. The dependent variable was a dummy variable of the current employment situation, which received the value of 1 if the individual worked last week and 0 otherwise.

### The model

$${\text{logit}}\left( {{\text{p}}_{\text{i}} } \right) = \log \left( {\frac{{{\text{p}}_{\text{i}} }}{{1 - {\text{p}}_{\text{i}} }}} \right) =\upbeta\underset{\raise0.3em\hbox{$\smash{\scriptscriptstyle-}$}}{\text{X}}_{\text{i}}$$

i—individual i, P_i_—the chances that individual i will have a full or part time job (at the time of the survey). $$\underset{\raise0.3em\hbox{$\smash{\scriptscriptstyle-}$}}{\text{X}}_{\text{i}}$$—vector of explanatory variables of individual i. Each of the variables in vector $$\underset{\raise0.3em\hbox{$\smash{\scriptscriptstyle-}$}}{X}_{i}$$ was defined as a dummy variable with the value of 1 or 0. β—vector of marginal addition to the log of the odds ratio. For example, if the explanatory variable was the log of 13 years or more of schooling, then the log odds ratio refers to the marginal addition of 13 years of education to the chances of being employed, compared with 12 years of education or less.

The regression allowed us to predict the probability of an individual finding a job. The dependent variable was the natural base log of the probability ratio P divided by (1 − P) that a particular individual would find a job. The odds ratio from the regression answers the question of how much more likely it is that an individual will find a job if he or she has certain characteristics. The importance of the probability analysis is the consideration of the marginal contribution of each feature to the probability of finding a job.

### The sample

We used data gathered from the 2011 Labor Force Survey^5^ of the Israeli Central Bureau of Statistics (CBS),^6^ which is a major survey conducted annually among households. The survey follows the development of the labor force in Israel, its size and characteristics, as well as the extent of unemployment and other trends. Given our focus on working age individuals, we excluded all of the respondents under the age of 18 or over the age of 59. The data sample includes only the Jewish population, because structural problems in the non-Jewish sector made it difficult to estimate this sector using the existing data only. The sample does not include the ultra-Orthodox population because of their special characteristics, particularly the limited involvement of men in this population in the labor market.

The base population is individuals who did not work at all during the past year or worked less than 9 months last year (meaning that they worked but were unemployed at least part of last year). To determine whether they managed to find work after 1 year of unemployment, we used the question on the ICBS questionnaire, “Did you work last week?” We used the answer to this question to distinguish between those who had succeeded in finding a job and those who did not. The data include individuals who were out of the labor force^7^ at the time of the survey, but exclude those who were not working for medical reasons (illness, disability or other medical restrictions) or due to their mandatory military service.^8^

### Data and variables

The survey contains 104,055 respondents, but after omitting all of the respondents under the age of 18 or above 59, those who were outside the labor force for medical reasons or due to mandatory military service, non-Jews, the ultra-Orthodox, and those who worked more than 9 months last year, the sample includes 13,494 individuals (the base population). Of these, 9409 are individuals who had not managed to find work, and 4085 are individuals who were employed when the survey was conducted.

The participants’ ages range between 18 and 59, with the average age being 33.07 (SD 12.88) and the median age being 29. 40.8% are males; 43.5% have an academic education; 52.5% are single, and 53.5% of the respondents have no children under 17.

### Dependent and independent variables

While previous studies have assessed the probability of being unemployed in the general population, our study examines a more specific case: the probability of unemployed individuals finding a job. Therefore, we use the same explanatory variables that have been used in similar studies conducted in Israel (Brander et al. 2002; Flug and Kassir 2001), which were also based on an income survey and the Labor Force Survey of the Central Bureau of Statistics.

### The dependent variable—being employed

According to the definition of the CBS, employed persons are those who worked at least 1 h during a given week for pay, profit or other compensation.

### Independent variables

#### Age

We divided the population into sub-groups of age intervals: 18–24, 25–29, 30–44, 45–54 and 55–59, according to the sub-groups provided by the CBS. We then assigned a special dummy variable to each group—except the 30–44 sub-group, which is considered as the base group. Age is measured as a dummy variable, and is codded as 1 if the individual belongs to the age group, and 0 otherwise. Age appears in the regression results as a variable in and of itself. Its significance is the marginal contribution of each age group to the probability of finding work relative to the base group (ages 30–44), and also as an interaction variable.

#### Gender

This variable is codded as 1 if the individual is female and 0 otherwise. Gender also appears in the interaction with age.

#### Marital status

Two dummy variables are used: one for married respondents and one for those who are divorced or widowed. In accordance with the practice of the CBS, we combined the divorced and the widowed into one variable. This variable is a dummy variable that is codded as 1 if the individual belongs to the appropriate group (divorced/widowed or married) and 0 otherwise. The base group is those who are single.

#### Education

This variable is codded as 1 if the individual has 13 or more years of schooling, and 0 otherwise. The variable also appears in interactions between it and the age variable.

#### Vocational education

This variable is codded as 1 if the individual has a secondary school diploma that is not an academic degree or another diploma, and 0 otherwise.

#### Academic education

This variable is codded as 1 if the individual has any university degree (bachelors, masters or Ph.D.) and 0 otherwise.

#### Children

In accordance with similar studies that examined the probability of employment in Israel (Brander et al. 2002), we define children as those up to age 17. This variable is a dummy variable that is codded as 1 if the respondents have children under the age of 17, and 0 otherwise.

#### Ethnicity

This variable is codded as 1 if the individual was born in an Arabic-speaking country, in an African country other than South Africa, or in an Asian country, or was born in Israel but had a father who was born in one of these countries. Israel generally refers to such individuals as Mizrahim. Respondents who were not Mizrahim received a value of 0. The base group in our study are men aged 30–44 who are not Mizrahim.

We also assessed the interactions between the variables. For example, the interaction between age and the number of years of schooling is the contribution of education (i.e., 13 years of schooling) to the probability of finding a job for every age group separately relative to the situation of having less education (i.e., 12 years of education). The interaction between age and gender is the contribution of gender (i.e., being a female respondent) to the probability of finding a job for each age group separately relative to being a man.

## Results

To demonstrate the differences between old and young individuals in their chances of finding a job, we computed the rates of those who managed to find a job relative to all of the respondents in the sample. Table 1 shows that the rate of those who found a job declines with age. For example, 36% of the men age 30–44 found a job, but those rates drop to 29% at the age of 45–54 and decline again to 17% at the age of 55–59. As for women, 31% of them aged 30–44 found a job, but those rates drop to 20% at the age of 45–54 and decline again to 9% at the age of 55–59.

**Table 1:** The rate of males and females who found a job (out of the entire group)

	Males (%)	Females (%)	Total (%)
18–24	33	35	34
25–29	34	36	35
30–44	36	31	33
45–54	29	20	23
55–59	17	9	12
Total	32	29	30

In an attempt to determine the role of education in finding employment, we created Model 1 and Model 2, which differ only in terms of how we defined education. In Model 1 the sample is divided into two groups: those with up to 12 years of schooling (the base group) and those with 13 or more years of schooling. In Model 2 there are three sub-groups: those with a university degree, those who have a vocational education, and the base group that has only a high school degree.

Table 2 shows that the probability of a young person (age 18–24) getting a job is larger than that of an individual aged 30–44 who belongs to the base group (the coefficient of the dummy variable “age 18–24” is significant and positive). Similarly, individuals who are older than 45 are less likely than those in the base group to find work.

**Table 2:** Chances of being employed—entire sample

Variable	Model 1	Model 2
	Coefficient (std. error)	Coefficient (std. error)
C	− 0.773*** (0.060)	− 0.833*** (0.054)
Gender	− 0.228*** (0.073)	− 0.230*** (0.071)
Ages 18–24	0.237*** (0.073)	0.157** (0.068)
Ages 45–54	− 0.201* (0.107)	− 0.210** (0.101)
Ages 55–59	− 1.051*** (0.132)	− 0.948*** (0.140)
Educated	0.554*** (0.067)	
Vocational education		0.329*** (0.073)
Academic education		0.852*** (0.079)
Children	− 0.184*** (0.041)	− 0.164*** (0.041)
Ethnicity	0.146*** (0.042)	0.166*** (0.042)
Gender × ages 18–24	0.374*** (0.095)	0.305*** (0.093)
Gender × ages 25–29	0.419*** (0.099)	0.285*** (0.089)
Gender × ages 45–54	− 0.284** (0.136)	− 0.296** (0.134)
Gender × ages 55–59	− 0.575*** (0.185)	− 0.561*** (0.184)
Educated × ages 18–24	− 1.031*** (0.094)	
Educated × ages 25–29	− 0.469*** (0.098)	
Educated × ages 45–54	− 0.361*** (0.134)	
Vocational education × ages 18–24		− 0.274* (0.151)
Academic education × ages 18–24		− 0.535*** (0.202)
Academic education × ages 25–29		− 0.236* (0.129)
Academic education × ages 45–54		− 0.473*** (0.161)
Academic education × ages 55–59		− 0.358* (0.204)

Dependent variable: being employed

Included observations: 13,495

* *p* < 0.1, ** *p* < 0.05, *** *p* < 0.01

Women aged 30–44 are less likely to be employed than men in the same age group. Additionally, when we compare women aged 18–24 to women aged 30–44, we see that the chances of the latter being employed are lower. Older women (45+) are much less likely than men of the same age group to find work. Additionally, having children under the age of 17 at home reduces the probability of finding a job.

A university education increases the probability of being employed for both men and women aged 30–44. Furthermore, for older people (55+) an academic education reduces the negative effect of age on the probability of being employed. While a vocational education increases the likelihood of finding a job for those aged 30–44, such a qualification has no significant impact on the prospects of older people.

Interestingly, being a Mizrahi Jew increases the probability of being employed.

In addition, we estimated the models separately twice—for the male and for the female population. For male and female, the probability of an unemployed individual finding a job declines with age.

Analyzing the male population (Table 3) reveals that those aged 18–24 are more likely than the base group (ages 30–44) to find a job. However, the significance level is relatively low, and in Model 2, this variable is not significant at all. Those 45 and older are less likely than the base group (ages 30–44) to find a job. Married men are more likely than single men to be employed. However, divorced and widowed men are less likely than single men to find a job. For men, the presence in their household of children under the age of 17 further reduces the probability of their being employed. Mizrahi men aged 18–24 are more likely to be employed than men of the same age who are from other regions.

**Table 3:** Chances of being employed—males and females separately

	Males	Females		
Included observations	5508	7986		

Variable	Model 1	Model 2	Model 1	Model 2
	Coefficient (std. error)	Coefficient (std. error)	Coefficient (std. error)	Coefficient (std. error)
C	− 0.744*** (0.073)	− 0.733*** (0.044)	− 0.766*** (0.073)	− 0.869*** (0.071)
Married	0.422*** (0.093)	0.381*** (0.083)	− 0.183*** (0.068)	− 0.252*** (0.069)
Divorced/widowed	− 0.321* (0.164)	− 0.326** (0.159)		
Ages 18–24	0.167* (0.094)		0.446*** (0.084)	0.286*** (0.073)
Ages 45–54	− 0.245** (0.121)	− 0.376*** (0.099)	− 0.670*** (0.089)	− 0.614*** (0.089)
Ages 55–59	− 1.122*** (0.137)	− 1.103*** (0.136)	− 1.645*** (0.133)	− 1.620*** (0.132)
Educated	0.268** (0.106)		0.546*** (0.072)	
Educated × ages 18–24	− 0.726*** (0.150)		− 1.042*** (0.112)	
Educated × ages 25–29	− 0.292** (0.133)		− 0.204** (0.101)	
Educated × ages 45–54	− 0.406* (0.212)			
Vocational education		0.360*** (0.123)		0.263*** (0.088)
Academic education		0.182** (0.90)		0.922*** (0.073)
Children	− 0.215*** (0.069)	− 0.176*** (0.067)	− 0.209*** (0.060)	− 0.168*** (0.059)
Ethnicity				0.115** (0.059)
Ethnicity × ages 18–24	0.313*** (0.106)	0.337*** (0.100)		
Ethnicity × ages 25–29			0.411*** (0.128)	0.289** (0.132)
Vocational education × ages 18–24		− 0.602** (0.264)		
Vocational education × ages 25–29				0.381** (0.183)
Academic education × ages 18–24				− 0.484** (0.242)

Dependent variable: being employed

* *p* < 0.1, ** *p* < 0.05, *** *p* < 0.01

Table 3 illustrates that educated men are more likely to find work than those who are not. However, in Model 1, at the ages 18–29 and 45–54, the probability of finding a job for educated men is less than that of uneducated males. Among younger workers, this might be due to excess supply—the share of academic degree owners has risen, in contrast to almost no change in the overall share of individuals receiving some other post-secondary certificate (Fuchs 2015). Among older job seeking men, this might be due to the fact that the increase in employment among men during 2002–2010 occurred mainly in part-time jobs (Bank of Israel 2011). In Model 2, men with an academic or vocational education have a better chance of finding a job, but at the group age of 18–24, those with a vocational education are less likely to find a job compared to those without a vocational education. The reason might be the lack of experience of young workers (18–24), experience that is particularly needed in jobs that require vocational education (Salvisberg and Sacchi 2014).

Analyzing the female population (Table 3) reveals that women between 18 and 24 are more likely to be employed than those who are 30–44, and those who are 45–59 are less likely to be employed than those who are 30–44. The probability of finding a job for women at the age of 25 to 29 is not significantly different from the probability of the base group (women ages 30–44).

Married women are less likely than single women to be employed. Women who have children under the age of 17 are less likely to be employed than women who do not have dependents that age. According to Model 2, Mizrahi women are more likely to be employed compared to women from other regions. According to both models, women originally from Asia or Africa ages 25–29 have a better chance of being employed than women the same age from other regions. Future research should examine this finding in depth to understand it.

With regard to education, in Model 1 (Table 3), where we divided the respondents simply on the question of whether they had a post-high school education, women who were educated were more likely to find work than those who were not. However, in the 18–29 age categories, educated women were less likely to find a job compared to uneducated women, probably due to the same reason cited above for men in the same age group—the inflation of academic degrees (Fuchs 2015). These findings become more nuanced when we consider the results of Model 2. There, women with an academic or vocational education have a better chance of finding a job, but at the ages of 18–24 those with an academic education are less likely to find a job than those without an academic education. Finally, at the ages of 25–29, those with a vocational education have a better chance of finding a job than those without a vocational education, due to the stagnation in the overall share of individuals receiving post-secondary certificate (Fuchs 2015).

Thus, based on the results in Table 3, we can draw several conclusions. First, the effect of aging on women is more severe than the impact on men. In addition, the “marriage premium” is positive for men and negative for women. Divorced or widowed men lose their “marriage premium”. Finally, having children at home has a negative effect on both men and women—almost at the same magnitude.

## Unemployment as a function of the business cycle

To determine whether unemployment of young workers is caused by the business cycle, we examined the unemployment figures in 34 OECD countries in 2007–2009, years of economic crisis, and in 2009–2011, years of recovery and economic growth. For each country, we considered the data on unemployment among young workers (15–24) and older adults (55–64) and calculated the difference between 2009 and 2007 and between 2011 and 2009 for both groups. The data were taken from OECD publications and included information about the growth rates from 2007 to 2011. Our assessment of unemployment rates in 34 OECD countries reveals that the average rate of youth unemployment in 2007 was 13.4%, compared to 18.9% in 2011, so the delta of youth unemployment before and after the economic crisis was 5.55. The average rate of adult unemployment in 2007 was 4% compared to 5.8% in 2011, so the delta for adults was 1.88. Both of the differences are significantly different from zero, and the delta for young people is significantly larger than the delta for adults. These results indicate that among young people (15–24), the increase in unemployment due to the crisis was very large.

An OLS model of the reduced form was estimated to determine whether unemployment is a function of the business cycle, which is represented by the growth rate. The variables GR2007, GR2009 and GR2011 are the rate of GDP growth in 2007, 2009 and 2011 respectively (Appendix). The explanatory variable is either GR2009 minus GR2007 or GR2011 minus GR2009. In both periods, 2007–2009 and 2009–2011, the coefficient of the change in growth rates is negative and significant for young people, but insignificant for adults. Thus, it seems that the unemployment rates of young people are affected by the business cycle, but those of older workers are not. In a time of recession (2007–2009), unemployment among young individuals increases whereas for older individuals the increase in unemployment is not significant. In recovery periods (2009–2011), unemployment among young individuals declines, whereas the drop in unemployment among older individuals is not significant (Table 4).

**Table 4:** Unemployment rate as a function of the business cycle

Variables	The growth of youth unemployment 2007–2009		The growth of older unemployment 2007–2009	
	Coefficient	Std. error	Coefficient	Std. error
Constant	0.553	1.635	2.539**	1.144
GR 2009–2007	− 0.530***	0.181	− 0.184	0.125
Adjusted R squared	0.187		0.035	

Variables	The growth of youth unemployment 2009–2011		The growth of older unemployment 2009–2011	
	Coefficient	Std. error	Coefficient	Std. error
Constant	4.486***	1.191	− 1.581***	0.514
GR 2011–2009	− 0.602***	0.155	− 0.084	0.066
Adjusted R squared	0.299		0.019	

Dependent variable: the increase in the unemployment rate between 2007 and 2009, and between 2009 and 2011

* *p* < 0.1, ** *p* < 0.05, *** *p* < 0.01

## Summary and conclusions

The purpose of this paper was to show that while the unemployment rates of young workers are higher than those of older workers, the data alone do not necessarily tell the whole story. Our findings confirm our first hypothesis, that the high unemployment rate of young people stems mainly from the characteristics of the labor market and less from their personal attributes. Using data from Israel and 34 OECD countries, we demonstrated that a country’s growth rate is the main factor that determines youth unemployment. However, the GDP rate of growth cannot explain adult unemployment. Our results also support our second hypothesis, that the difficulties faced by unemployed older workers when searching for a job are more a function of their age than the overall business environment.

Indeed, one limitation of the study is the fact that we could not follow individuals over time and capture individual changes. We analyze a sample of those who have been unemployed in the previous year and then analyze the probability of being employed in the subsequent year but cannot take into account people could have found a job in between which they already lost again. Yet, in this sample we could isolate and analyze those who did not work last year and look at their employment status in the present. By doing so, we found out that the rate of those who found a job declines with age, and that the difficulties faced by unemployed older workers stems mainly from their age.

To solve both of these problems, youth unemployment and older workers unemployment, countries need to adopt different methods. Creating more jobs will help young people enter the labor market. Creating differential levels for the minimum wage and supplementing the income of older workers with earned income tax credits will help older people re-enter the job market.

Further research may explore the effect of structural and institutional differences which can also determine individual unemployment vs. employment among different age groups.

In addition to presenting a theory about the factors that affect the differences in employment opportunities for young people and those over 45, the main contribution of this paper is demonstrating the validity of our contention that it is age specifically that works to keep older people out of the job market, whereas it is the business cycle that has a deleterious effect on the job prospects of younger people. Given these differences, these two sectors of unemployment require different approaches for solving their employment problems. The common wisdom maintains that the high level of youth unemployment requires policy makers to focus on programs targeting younger unemployed individuals. However, we argue that given the results of our study, policy makers must adopt two different strategies to dealing with unemployment in these two groups.

### Policy implications

In order to cope with the problem of youth unemployment, we must create more jobs. When the recession ends in Portugal and Spain, the problem of youth unemployment should be alleviated. Since there is no discrimination against young people—evidenced by the fact that when the aggregate level of economic activity and the level of adult employment are high, youth employment is also high—creating more jobs in general by enhancing economic growth should improve the employment rates of young workers.

In contrast, the issue of adult unemployment requires a different solution due to the fact that their chances of finding a job are related specifically to their age. One solution might be a differential minimum wage for older and younger individuals and earned income tax credits (EITC)^9^ for older individuals, as Malul and Luski (2009) suggested.

According to this solution, the government should reduce the minimum wage for older individuals. As a complementary policy and in order to avoid differences in wages between older and younger individuals, the former would receive an earned income tax credit so that their minimum wage together with their EITC would be equal to the minimum wage of younger individuals. Earned income tax credits could increase employment among older workers while increasing their income. For older workers, EITCs are more effective than a minimum wage both in terms of employment and income. Such policies of a differential minimum wage plus an EITC can help older adults and constitute a kind of social safety net for them. Imposing a higher minimum wage exclusively for younger individuals may be beneficial in encouraging them to seek more education.

Young workers who face layoffs as a result of their high minimum wage (Kalenkoski and Lacombe 2008) may choose to increase their investment in their human capital (Nawakitphaitoon 2014). The ability of young workers to improve their professional level protects them against the unemployment that might result from a higher minimum wage (Malul and Luski 2009). For older workers, if the minimum wage is higher than their productivity, they will be unemployed. This will be true even if their productivity is higher than the value of their leisure. Such a situation might result in an inefficient allocation between work and leisure for this group. One way to fix this inefficient allocation without reducing the wages of older individuals is to use the EITC, which is actually a subsidy for this group. This social policy might prompt employers to substitute older workers with a lower minimum wage for more expensive younger workers, making it possible for traditional factories to continue their domestic production. However, a necessary condition for this suggestion to work is the availability of efficient systems of training and learning. Axelrad et al. (2013) provided another justification for subsidizing the work of older individuals. They found that stereotypes about older workers might lead to a distorted allocation of the labor force. Subsidizing the work of older workers might correct this distortion. Ultimately, however, policy makers must understand that they must implement two different approaches to dealing with the problems of unemployment among young people and in the older population.

## Appendix

See Table 5.

**Table 5:** Gross domestic product, volume, annual growth rates in percentage. Source: National Accounts at a Glance 2014, OECD, 2014. http://www.oecd-ilibrary.org/economics/national-accounts-at-a-glance-2014_na_glance-2014-en

	Growth 2007	Growth 2009	Growth 2010	Growth 2011
Australia	3.7	2.0	2.2	3.6
Austria	3.7	− 3.8	1.8	2.8
Belgium	2.9	− 2.8	2.3	1.8
Canada	2.2	− 2.8	3.2	2.5
Chile	5.2	− 1.0	5.8	5.9
Czech Republic	5.7	− 4.5	2.5	1.8
Denmark	1.6	− 5.7	1.4	1.1
Estonia	7.5	− 14.1	2.6	9.6
Finland	5.3	− 8.5	3.4	2.7
France	2.3	− 3.1	1.7	2.0
Germany	3.3	− 5.1	4.0	3.3
Greece	3.5	− 3.1	− 4.9	− 7.1
Hungary	0.1	− 6.8	1.1	1.6
Iceland	6.0	− 6.6	− 4.1	2.7
Ireland	5.0	− 6.4	− 1.1	2.2
Israel	5.9	1.1	5.0	4.6
Italy	1.7	− 5.5	1.7	0.5
Japan	2.2	− 5.5	4.7	− 0.6
Korea	5.1	0.3	6.3	3.7
Luxembourg	6.6	− 5.6	3.1	1.9
Mexico	3.4	− 6.0	5.3	3.9
Netherlands	3.9	− 3.7	1.5	0.9
New Zealand	3.5	1.5	0.2	2.2
Norway	2.7	− 1.6	0.5	1.3
Poland	6.8	1.6	3.9	4.5
Portugal	2.4	− 2.9	1.9	− 1.3
Slovak Republic	10.5	− 4.9	4.4	3.0
Slovenia	7.0	− 7.9	1.3	0.7
Spain	3.5	− 3.8	-0.2	0.1
Sweden	3.3	− 5.0	6.6	2.9
Switzerland	3.8	− 1.9	3.0	1.8
Turkey	4.7	− 4.8	9.2	8.8
United Kingdom	3.4	− 5.2	1.7	1.1
United States	1.8	− 2.8	2.5	1.8

## Footnotes

1 For example, in the US, the UK and Portugal, we witnessed higher rates of growth during late 1990 s and lower rates of youth unemployment compared to 2011.

2 Bank of Israel Annual Report—2013, http://www.boi.org.il/en/NewsAndPublications/RegularPublications/Research%20Department%20Publications/BankIsraelAnnualReport/Annual%20Report-2013/p5-2013e.pdf.

3 Bank of Israel Annual Report—2013, http://www.boi.org.il/en/NewsAndPublications/RegularPublications/Research%20Department%20Publications/BankIsraelAnnualReport/Annual%20Report-2013/p5-2013e.pdf.

4 http://www.boi.org.il/en/NewsAndPublications/RegularPublications/Research%20Department%20Publications/RecentEconomicDevelopments/develop136e.pdf.

5 The Labor Force Survey is a major survey conducted by the Israeli Central Bureau of Statistics among households nationwide. The survey follows the development of the labor force in Israel, its size and characteristics, as well as the extent of unemployment and other trends. The publication contains detailed data on labor force characteristics such as their age, years of schooling, type of school last attended, and immigration status. It is also a source of information on living conditions, mobility in employment, and many other topics.

6 The survey population is the permanent (de jure) population of Israel aged 15 and over. For more details see: http://www.cbs.gov.il/publications13/1504/pdf/intro04_e.pdf.

7 When we looked at those who had not managed to find a job at the time of the survey, we included all individuals who were not working, regardless of whether they were discouraged workers, volunteers or had other reasons. As long as they are not out of the labor force due to medical reasons or their mandatory military service, we classified them as "did not manage to find a job."

8 Until 2012, active soldiers were considered outside the labor force in the samples of the CBS.

9 EITC is a refundable tax credit for low to moderate income working individuals and couples.
